# Fluctuating Roles of Matrix Metalloproteinase-9 in Oral Squamous Cell Carcinoma

**DOI:** 10.1155/2013/920595

**Published:** 2013-01-08

**Authors:** Suvi-Tuuli Vilen, Tuula Salo, Timo Sorsa, Pia Nyberg

**Affiliations:** ^1^Biomedicum Helsinki, Institute of Dentistry, Research Laboratory, University of Helsinki, P.O. Box 63, Haartmaninkatu 8, 00014 Helsinki, Finland; ^2^Department of Diagnostics and Oral Medicine, Institute of Dentistry and the Oulu Center for Cell-Matrix Research, University of Oulu, P.O. Box 5281, 90014 Oulu, Finland; ^3^Oulu University Hospital, Oulu, Finland; ^4^Department of Oral and Maxillofacial Diseases, Helsinki University Hospital, Helsinki, Finland

## Abstract

One hallmark of cancer is the degradation of the extracellular matrix (ECM), which is caused by proteinases. In oral cancers, matrix metalloproteinases (MMPs), especially MMP-9, are associated with this degradation. MMPs break down the ECM allowing cancer to spread; they also release various factors from their cryptic sites, including cytokines. These factors modulate cell behavior and enhance cancer progression by regulating angiogenesis, migration, proliferation, and invasion. The development of early metastases is typical for oral cancer, and increased MMP-9 expression is associated with a poor disease prognosis. However, many studies fail to relate MMP-9 expression with metastasis formation. Contrary to earlier models, recent studies show that MMP-9 plays a protective role in oral cancers. Therefore, the role of MMP-9 is complicated and may fluctuate throughout the different types and stages of oral cancers.

## 1. Introduction

Oral cancer is one of the ten most common cancers worldwide. Nearly 3% of all cancer cases are oral cancers; they are more common in men than in women; and two-thirds of oral cancer cases occur in developing countries [1, 2]. One important hallmark of cancer progression is the degradation of the extracellular matrix (ECM), which allows cancer cells to invade the surrounding tissue. Matrix metalloproteinases (MMPs) are zinc-dependent endopeptidases that efficiently degrade the components of the ECM and basement membranes (BM). MMPs also release cytokines, chemokines, and growth factors from their proforms or their cryptic sites [3–6]. To date, at least 24 distinct MMP genes have been identified in humans. MMPs are classified according to their substrate specificities: gelatinases, collagenases, matrilysins, and stromelysins. The structures of all MMPs include an N-terminal signal peptide that directs the protein to either the plasma membrane insertion or to the secretory pathway; its prodomain confers its latency, and its catalytic domain has a zinc atom in its active site. MMPs are either anchored in the membrane or secreted, primarily as latent proforms that require activation before becoming catalytically competent [7–9]. Two different soluble gelatinases have been identified: gelatinase A, 72 kDa (MMP-2), and gelatinase B, 92 kDa (MMP-9). Both contain a collagen-binding domain within their catalytic domain, distinguishing them from other MMPs. A more detailed structure of these enzymes is described in a review by Björklund and Koivunen [10]. 

## 2. Activation of MMP-9

Typically, gelatinases are secreted as inactive zymogens that become activated extracellularly. The most relevant natural activators of proMMP-9 are unknown, but proMMP is activated through a few different mechanisms, including proteolytic activation, where the prodomain is cleaved yielding an active enzyme. Latent MMP-9 can be activated by MMP-3, which cleaves proMMP-9 at multiple sites: the first cleavage site is Glu^59^-Met^60^; the second is Arg^106^-Phe^107^ [11]. In contrast, MMP-26 activates MMP-9 by cleaving at Ala^93^-Met^94^ [12]. Previous studies have also demonstrated that enterokinase, a membrane-bound serine protease, cleaves proMMP-9 at Lys^65^-Ser^66^ [13] and that trypsin-2 activates proMMP-9 at very low molar ratios, 1 : 1000. The peptide bond can also be cleaved at Arg^87^-Phe^88^ [14]. Other known proteolytic activators are plasmin, chymotrypsin-like proteinase, MMP-2, MMP-7, MMP-10, and MMP-13 [15–20]. There are other identified activation mechanisms for MMP-9: oxidation by reactive oxygen species, S-nitrosylation, and allosteric activation, which occurs when proMMP-9 is bound to either a gelatin or type IV collagen [21–23]. In an invasive tongue squamous cell carcinoma cell line (HSC-3), MMP-9 is colocalized with trypsin-2 in intracellular vesicles [13]. This intracellular activation may be an alternative activation mechanism for proMMPs in oral cancers. Similar intracellular vesicle transports for MMP-9 are also found in melanoma cells and in ovarian cancer ascites [24, 25]. In oral squamous cell carcinoma (OSCC), the activation level of MMP-9 may be associated with a shortened disease-free survival and a high metastatic frequency [26].

## 3. Inhibitors of MMP-9

Tissue inhibitors of metalloproteinases (TIMPs) are specific endogenous inhibitors of MMPs, which bind MMPs in a 1 : 1 stoichiometry. Four different TIMPs have been identified: TIMP-1, TIMP-2, TIMP-3, and TIMP-4 [27]; they all inhibit MMP-9 *in vitro *[28–31]. The role of TIMPs in OSCC is well-studied and is the focus of a recent review by Garćia et al. [32]. After the role of MMPs in cancer invasion and metastasis formation was recognized, researchers began developing synthetic inhibitors. The first generation of peptidomimetic MMPs, batimastat (BB94) and ilomastat (GM-6001), mimicked the structure of collagen and reversibly bound the active site of MMPs to inhibit MMP activity [33, 34]. Next, Marimastat (BB-2516), a second generation of MMP inhibitors, was developed. However, all of these broad-spectrum inhibitors failed in clinical trials due to their side effects and their lack of efficacy [35–37], which led to the development of more selective matrix metalloproteinase inhibitors (MMPIs). We developed the first gelatinase-specific MMPI CTTHWGFTLC peptide, which inhibited the invasion of ovarian carcinoma, breast carcinoma, fibrosarcoma, Kaposi's sarcoma, and melanoma cell lines, *in vitro*; it also increased the survival of human tumor xenografts [38]. Since then, we have developed a cyclic gelatinase-specific inhibitor GRENYHGCTTHWGFTLC peptide, which inhibits MMP-9 activation and activity, the growth of human tongue cancer cell xenografts, and angiogenesis in nude mice [39]. The synthetic MMPIs that inhibit MMP-9 are listed in Table 1. 

## 4. MMP-9 in the Oral Microenvironment

In addition to carcinoma cells, cancers consist of tumor-associated stromal cells, which include fibroblasts, endothelial cells, leukocytes, macrophages, nerve cells, and adipocytes. During cancer progression, the cancer cells crosstalk with stromal components and their interactions are partially mediated by transmembrane receptors, which are expressed on cancer cells and stromal cells. Tumor-associated cells promote angiogenesis, inflammation, invasion, and ECM modeling through cell-cell contact and the production of growth factors, hormones, cytokines, and proteinases such as MMPs [56–58]. In OSCC tumors, MMP-9 is expressed in carcinoma and inflammatory cells around carcinoma islands. Meanwhile, MMP-2 is mainly found in carcinoma-associated fibroblasts (CAFs) [59, 60]. In an oral squamous cell carcinoma cell line SCC-25, CAFs increase the expression of MMP-9, *in vitro*, which is thought to occur via a fibronectin-integrin *α*v*β*6 pathway [61]. In the aggressive human tongue squamous cell carcinoma cell line HSC-3, MMP-2 was only found in its latent form, whereas MMP-9 was found in its active form [13]. MMP-9′s effect during HSC-3 cell line invasion was studied in a human organotypic model based on myoma tissue [62], which contains fibroblasts, smooth muscle cells, lymphocytes, macrophages, endothelial cells, and MMP-2, but not MMP-9 [62]. Therefore, this model is a better predictor of the *in vivo* tumor microenvironment compared with the commonly used rat tail-derived type I collagen and/or the mouse EHS sarcoma-derived Matrigel invasion assay. In the myoma organotypic invasion assay, after inhibiting gelatinase activity in HSC-3 cells using a specific gelatinase inhibitor CTTHWGFTLC [38], the tumor cells were surprisingly more invasive than in the control group (unpublished data). Mice bearing HSC-3 xenograft tumors treated with the gelatinase inhibitor CTTHWGFTLC had smaller primary tumors *in vivo* than the control group [39], but the inhibition of gelatinases did not affect local invasion or metastasis formation [63]. The ability of cancer cells to change their migration under certain circumstances from proteolytic to non-proteolytic, amoeboid type during protease-inhibitor treatment helps to explain the OSCC behaviors we observed. Thus, these cells change their shape and adapt to squeeze through tissue gaps without degrading the ECM [64]. MMP-9 may not be the only, or even the most important, proteolytic enzyme in the OSCC invasion process, but it may be important for indirect cell signaling by controlling the bioavailability and bioactivity of molecules that target specific receptors, which regulate cell growth, migration, inflammation, and angiogenesis [65–69].

## 5. The Role of MMP-9 in OSCC Invasion and Metastasis

MMP-9 is associated with the aggressive nature of many cancers, including OSCC [81–84], and this aggressive nature was thought to cause type IV collagen degradation, a main component of basement membranes [85]. To date, the spectrum of MMP-9 matrix substrates has significantly increased, and aside from substrates, which originate in the matrix, MMP-9 has other bioactive substrates that independently modulate carcinogenesis, such as the pro-transforming growth factor-*β*1 (TGF-*β*1) and the pro-tumor necrosis factor-*α* (TNF-*α*) [10, 86, 87]. MMP-9 has traditionally been associated with the aggressive nature of OSCC. However, in spite of increased MMP-9 expression levels, many researchers have presented contradictory results [70–72] (Table 2). For example, Guttman et al. [73] did not find a correlation between MMP-9 expression and the size of the primary tumor or the neck metastasis in tongue SCC patients. Meanwhile, another study reported that high levels of MMP-9 expression in OSCC patients were correlated with regional lymph node and/or distant metastases and a poor prognosis [74]. In addition, De Vicente et al. [78] showed that MMP-9 expression was not associated with clinical variables, such as tumor stage or recurrence rate. In a study conducted by Ikebe et al., gelatinolytic activity and increased expression of both MMP-2 and MMP-9 in OSCC tumors were related to the invasiveness, but not to the metastatic potential of OSCC tumors [75]. Finally, Kato and co-workers [76] showed that, although MMP-9 expression was high in OSCCs, the activated form:proform ratio was very low, while activated MMP-2s were elevated and associated with advanced stages of disease. These findings suggest that MMP-9 may not be a universal cancer progression promotion factor in OSCCs; instead, it may have fluctuating roles. 

## 6. MMP-9 in the Modulation of Cancer-Related Inflammation

Chronic inflammation is associated with epithelial cancers, and it differs from normal inflammation because it is not self-limiting. Cancer cells produce different cytokines that attract innate immune cells, such as mast cells, granulocytes, and macrophages. These innate immune cells then secrete interleukins, chemokines, reactive oxygen species, and MMPs that modulate angiogenesis, cell proliferation, tumor growth, and invasion [88–90]. In OSCCs, the level of a multifunctional cytokine, transforming growth factor-*β*1, is upregulated, which leads to the enhanced expression of snail. Snail is a transcription factor that increases MMP-9 expression and triggers an epithelial-mesenchymal transition (EMT); then, carcinoma cells change their morphology, reduce their intercellular and cell-matrix adhesions, and increase their motility [91, 92]. Interestingly, the inactive form of TGF-*β*1 is activated by MMP-9 [93]. Many other cytokines are also substrates for MMP-9, including TNF-*α*, CXCL1, CXCL4, CXCL7, CXCL8, and interleukin-1*β* [65–67]. Interleukin-1*β* is secreted by tumor cells and induces the expression of lipocalin 2 [94, 95]. The plasma levels of lipocalin 2, MMP-9 and the lipocalin 2/MMP-9 complex are associated with more advanced clinical stages and/or tumor sizes in OSCC patients. Interestingly, MMP-9 levels are not correlated with either lymph nodes or distant metastases [77]. Chemokine CXCL8 can induce the release of MMP-9 from tertiary neutrophil granules, and increased CXCL8 expression is associated with OSCC. The CXCL8 expressed in tumor cells is also secreted by OSCC cell lines, and CXCL8 mRNA expression is enhanced by the addition of TNF-*α* and IL- 1*β*. CXCL8 from OSCC cell lines increases cell migration, induces invasion, and increases the expression of MMP-7. However, it does not have an effect on MMP-9 expression. Therefore, CXCL8-induced expression of MMP-9 may be cell-type specific [96–98]. The chemokine receptor, CXCR4, modulates the invasion of OSCCs by regulating MMP-9 expression. In patients, this expression correlates with lymph node metastasis and MMP-9 expression [99, 100]. Cyclo-oxygenase (COX)-2 is an enzyme that converts arachidonic acid into pro-inflammatory prostanoids. COX-2 has been implicated in carcinogenesis and its mRNA expression is nearly 150-fold greater in head and neck squamous cell carcinomas compared with normal oral mucosa. Using a selective COX-2 inhibitor decreases MMP-9 and MMP-2 expression and suppresses the proliferation and invasion of OSCCs [101, 102]. In fact, MMP-9 has many links to the cancer-related inflammation observed in OSCCs, but MMP-9′s specific role in this process remains unclear. 

## 7. MMP-9 in the Regulation of Angiogenesis

Low oxygen levels, or hypoxia, are typical in solid tumors that grow 1-2 mm^3^ without vascularization. Angiogenesis, the formation of new blood vessels, is required to bring nutrition and oxygen to cells, remove metabolic waste, and support larger tumor growth. Not only is angiogenesis associated with tumor growth, it is also related to the development of metastases in OSCCs [103–107]. This process is initiated by vascular endothelial growth factor (VEGF), an angiogenic cytokine. The effect of VEGF is mediated by vascular endothelial growth factor receptors. The hypoxic conditions stabilize hypoxia-inducible factors (HIFs) that bind to the VEGF promoter, which causes upregulation of VEGF and increases the expression of VEGFR-receptor-1 in endothelial cells, cancer cells and tumor-associated cells, such as macrophages [105, 108–111]. In OSCCs, overexpression of HIF-1*α* is associated with a poor patient outcome [112]. Increased tumor hypoxia is also associated with increased MMP-2 and MMP-9 activity [113].

**Figure 1:**
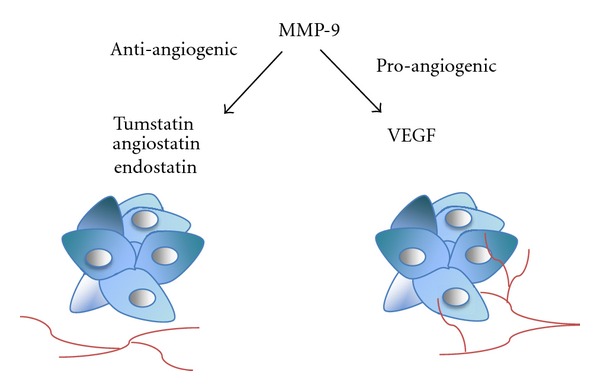
Effect of MMP-9 on angiogenesis in oral cancer. MMP-9 inhibits angiogenesis by releasing antiangiogenic factors from their precursors. MMP-9 enhances angiogenesis by releasing and activating VEGF from extracellular proteoglycans.

The regulation of angiogenesis is a very delicate balance between pro- and antiangiogenic factors, and it seems that MMP-9 plays a dual role in this process. It can act as a proangiogenic factor via VEGF regulation; as Hiratsuka et al. [114] demonstrated, primary tumors in premetastatic lungs induce MMP-9 expression in a VEGFR-1-dependent manner, which enhances the invasion of cancer cells and facilitates metastasis. MMP-9 also triggers the angiogenic switch by releasing VEGF [87]. MMP-9 and VEGF are expressed during invasive OSCCs of the tongue and in metastatic tumors that tend to express higher levels of VEGF and MMP-9, than nonmetastatic tumors [115]. In another study, increased VEGF expression was associated with a poor prognosis in OSCC patients, whereas MMP-9 expression levels had no correlation with patient outcomes [116]. This can be explained by the antiangiogenic role of MMP-9, which causes cleavage of type XVIII collagen, and leads to the release of endostatin, a potent inhibitor of angiogenesis and endothelial cell migration [68, 69]. OSCC primary tumors that do not metastasize have high endostatin levels compared with primary tumors that are associated with multiple metastatic lymph nodes. Full-length collagen XVIII expression levels are decreased in aggressive tumors [117]. Based on our study [118], collagen XVIII was expressed in mild oral epithelial dysplasias but was absent in the invasive fronts of OSCCs. Although MMP-9 expression was observed in these same samples, there was no correlation between MMP-9 and the stage of disease. Homer et al. [119] reported that in head and neck squamous cell carcinoma, the plasma levels of endostatin may predict tumor recurrence. Collagen XVIII and VEGF are both expressed at the same time in OSCCs, which further demonstrates that modulation of angiogenesis requires a delicate balance between angiogenic inhibitors and stimulators [109]. Moreover, we have demonstrated that endostatin also directly inhibits the invasion and intravasation of a human tongue SCC cell line and blocks the activity of proMMP-9, suggesting a feedback loop for MMP-9 regulation [120]. When mice bearing nasopharyngeal carcinoma tumors were treated with endostar (recombinant endostatin), the treatment led to a significant decrease in MMP-9 and VEGF expression levels and normalized the tumor vasculature [121]. Endostatin was not the only cryptic angiogenesis inhibitor liberated by MMP-9. Angiostatin, a fragment of plasminogen, also inhibits endothelial cell proliferation [86, 122]. Pozzi et al. [123] proved that decreased MMP-9 plasma levels in tumor-bearing mice also decreases angiostatin levels, which increases tumor vascularization and growth. Matsumoto et al. [124] observed that angiostatin-overexpressing SCC tumors in mice grew slower than the control tumors. Aside from endostatin and angiostatin production, MMP-9 is also associated with the proteolytic degradation of type IV collagen, which produces an angiogenesis inhibitor, tumstatin [125, 126]. These findings suggest that in cancer angiogenesis the variations in spatial and temporal MMP-9 expression may switch between two roles: from a proangiogenic to an antiangiogenic molecule (Figure 1).

## 8. Effect of Genetic and Environmental Factors on the Expression of MMP-9

The development and progression of OSCCs are a result of interactions between accumulating genetic alterations and environmental factors, such as alcohol, tobacco, viral infection, or chronic inflammation [127]. Despite newer cancer treatments, approximately 50% of patients die within 5 years of diagnosis [128]. This can be partially explained by the theory of oral field cancerization: an oral mucosa exposed to carcinogens, such as alcohol, causes multiple genetic abnormalities in the entire epithelium and increases the risk of developing several dysplastic lesions [129]. Polymorphisms in the *MMP9* gene allele are associated with an increased risk of developing the initial stages of oral cancer among patients without a family history of cancer and high smoking and/or alcohol use [130]. MMP-9 expression did not correlate with age, gender, tumor location, or smoking habits, whereas an association with tumor grade differentiation and alcohol consumption was observed [78]. MMP-9 was not expressed in the normal oral mucosa or dysplasia, whereas *in situ* carcinomas were weakly detectable. In OSCC, it was expressed in the same areas where collagen (IV) chain loss was observed at the invasive fronts, whereas in other studies, its overexpression was detected in 85% of oral dysplasias and in all of the oral cancer samples. The mRNA levels of MMP-9 were higher in oral dysplasia that progressed to oral SCC [131, 132]. Ogbureke et al. 2012 [133] proposed that MMP-9 expression at histologically negative surgical margins could predict OSCC recurrence. Interestingly, MMP-9 was absent from the margins of tumors ≤4 cm and only 10% of tumors without later node metastasis expressed it. MMP-9 expression alternates between different stages of malignant transformations. Different clinicopathological variables in OSCCs can partially be explained by viral infections in epithelial cells. The human papilloma virus (HPV) is associated with OSCCs, especially type 16. HPV16 transgenic mice, HPV16/MMP-9 +/+ and HPV16/MMP-9 +/− develop a similar incidence of SCC after 12 months of age, whereas HPV16/MMP-9 −/− mice have fewer tumors, but these tumors were more poorly differentiated than those in the other groups. The expression of HPV16 oncoproteins in human keratinocytes induced the upregulation of MMP-9 activity. At the same time, two natural MMP inhibitors were downregulated. One of them was the reversion-inducing cysteine-rich protein with Kazal motifs (RECK), which affects transcription, synthesis, activation, and the activity of MMPs; the other was TIMP-2 [134–136]. HPV16 infection may be one mechanism behind the contradictory expression of MMP-9 in OSCC patients; however, further studies are necessary to understand its significance.

## 9. Methods for MMP-9 Investigation in Oral Cancer

The most commonly used immunohistological analyses of MMP-9 expression can be misleading because most antibodies do not distinguish between the pro- and active forms of the protein. The total amount of protein expression does not necessarily mean that the enzyme is in an active form [137]. Gelatin zymography or *in situ* zymography [138, 139] are better methods to evaluate the level of gelatinase activity and would have provided more information in the studies referred to here (Table 2) [139]. Many other techniques can investigate the presence, amount and function of MMP-9. For example, *in situ* hybridization could define the location and number of cells that express MMP-9 mRNA in tissue sections, whereas the polymerase chain reaction (PCR) could detect the presence and amount of mRNA in tissues or cell extracts [140, 141]. More specific intracellular localization of MMP-9 could easily be achieved using confocal laser scanning microscopy, which enables a 3-dimensional definition of protein localization [13]. Enzyme-linked immunosorbent assays (ELISAs) could be used to determine the concentration of MMP-9 protein in serum and plasma samples; based on these studies, MMP-9 is a systemic biomarker that monitors the effectiveness of OSCC treatment [112, 141, 142]. Different blotting methods, Western, Northern, and Southern, reveal the expression of protein, RNA or DNA, respectively, in various samples [75, 143]. MMPIs, transfected cell lines, and knock-out animal models give more functional data about MMP-9 [144, 145]. Most likely, the variety of methodologies and the low sample sizes explain the high variation observed among the previous OSCC results. Therefore, more studies with larger well-documented clinical materials, using delicate methods, are necessary to determine the impact of MMP-9 in OSCC.

## 10. Conclusions

**Figure 2:**
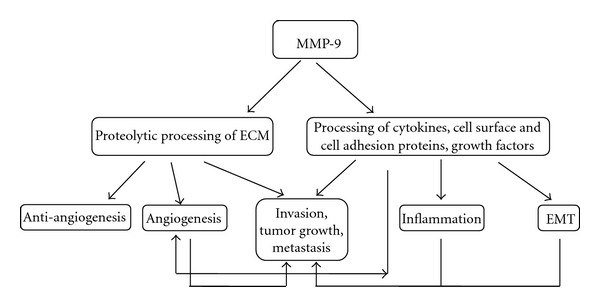
Schematic picture of the events that are modulated by MMP-9 in oral cancer. Proteolytic degradation of ECM components (including types III, IV, and V collagens, as well as gelatin) by MMP-9 facilitates carcinoma cell invasion and leads to the release of growth factors, such as VEGF, that enhance angiogenesis and tumor progression. At the same time anti-antiangiogenic endostatin, angiostatin, and tumstatin are released. Processing of proinflammatory chemokines (CXCL1, -4, -8, -9, -11, -12), proforms of cytokines (proTNF-*α*, proIL-1*β*), cell adhesion proteins such as intercellular adhesion molecule-1 triggers an inflammatory reaction and modulates transcription factors leading to an epithelial-to-mesenchymal transition and enhanced carcinogenesis.

Generally MMP-9 has been associated with aggressive head and neck cancers, but novel studies have shown that it acts as a protective molecule during carcinogenesis and metastasis. For example, in salivary gland myoepithelial carcinoma, MMP-9 expression predicts a better overall survival, and in regional metastases of head and neck cancers, *MMP9* gene expression was decreased [79, 146]. Similarly, the expression of MMP-9 is associated with a better outcome in breast- and colitis-associated carcinomas [147, 148]. Additionally, in oral cancer the role of MMP-9 was purely associated with the degradation of the ECM, which led to the enhancement of carcinoma cell invasion. However, the philosophy of oral carcinoma progression has become significantly more complicated; now, MMP-9 is known as a multifunctional modulator that is involved in very complex cell-signaling cascades (Figure 2). Therefore, in the case of MMP-9, one reason for the obvious failures of broad-spectrum and specific MMPIs in cancer treatment might be due to its fluctuating role in cancer, which not only affects carcinoma cells but also other cell populations. The tumor microenvironment matrix expresses and sequesters MMP-9. Taken together, our current knowledge of MMP-9 has been extended; it can act as either a carcinoma protector or promoter depending on the specific situation, which is related to patient characteristics, including the stage, grade, and location of the tumor.

## Figures and Tables

**Table 1:** Synthetic MMP-9 inhibitors.

Name	Type of drug	Specifity of the inhibition	Reference
Batimastat	Peptidomimetic	MMP-1, -2, -3, -7, -9	[40]
Marimastat	Peptidomimetic	Broad spectrum	[41]
CTTHWGFTLC	Peptidomimetic	MMP-2, -9	[38]
CGYGRFSPPC	Peptidomimetic	MMP-2, -9	[42]
CRVYGPYLLC	Peptidomimetic	MMP-2, -9	[42]
ABT-518	Peptidomimetic	MMP-2, -9	[43]
GRENYHGCTTHWGFTLC	Peptidomimetic	MMP-2, -9	[39]
Ilomastat	Peptidomimetic	Broad spectrum	[44]
Tanovastat	Nonpeptidomimetic	MMP-2, -3, -9	[45]
Prinovastat	Nonpeptidomimetic	MMP-2, -3, -7, -9, -13	[46]
BMS-275291	Nonpeptidomimetic	MMP-2, -9	[47]
Metastat (COL-3)	Chemically modified tetracycline	MMP-1, -2, -8, -9, -13	[48]
CMT-3	Chemically modified tetracycline	MMP-8, -9, -MT1-MMP	[49]
Doxycycline	Chemically modified tetracycline	MMP-1, -2, -3, -8, -9, -10, -13	[50]
SB-3CT	Reform proenzyme structure	MMP-2, -9	[51]
Bisphosphonates	Analogues of inorganic pyrophosphate	MMP-1, -2, -7, -9, MT1-, MT2-MMP	[52]
Chlorhexidine	Bisbiguanide antiseptic	MMP-2, -8, -9	[53]
Letrozole	Nonsteroidal inhibitor of aromatase	MMP-2, -9	[54]
PCK 3145	Synthetic peptide based on PSP94	MMP-9	[55]

**Table 2:** MMP-9 expression in oral cancer.

MMP-9 expression	Sample type	Lymph node metastasis	Outcome	MMP-9 activity	Method	Number of cases	References
High	Tissue	Yes	*	*	Immunohistochemistry	96	[70]
Yes	Tissue	Yes	*	*	Immunohistochemistry	61	[71]
High	Tissue	*	Shortened disease survival	High	Gelatin zymography,	44	[72]
Yes	Tissue	No	nc	*	Immunohistochemistry	23	[73]
High	Tissue	Yes	Poor	*	Immunohistochemistry	53	[74]
High	Tissue	No	*	High	Gelatin zymography, immunohistochemistry, western blot	57	[75]
High	Tissue	*	*	Low	Immunohistochemistry, gelatin zymography,	31	[76]
High	Plasma	No	*	*	ELISA	195	[77]
Yes	Tissue	No	nc	*	Immunohistochemistry	68	[78]
High	Tissue	*	Better survival	*	Immunohistochemistry	12	[79]
High	Tissue	Yes	Shortened disease survival	*	Immunohistochemistry	48	[80]

*not studied, nc: no correlation.

## References

[1] Jemal A, Bray F, Center MM, Ferlay J, Ward E, Forman D (2011). Global cancer statistics. *CA Cancer Journal for Clinicians*.

[2] Petersen PE (2009). Oral cancer prevention and control—the approach of the World Health Organization. *Oral Oncology*.

[3] Nagase H, Visse R, Murphy G (2006). Structure and function of matrix metalloproteinases and TIMPs. *Cardiovascular Research*.

[4] Butler GS, Overall CM (2009). Updated biological roles for matrix metalloproteinases and new “intracellular” substrates revealed by degradomics. *Biochemistry*.

[5] Klein T, Bischoff R (2011). Physiology and pathophysiology of matrix metalloproteases. *Amino Acids*.

[6] Giannelli G, Falk-Marzillier J, Schiraldi O, Stetler-Stevenson WG, Quaranta V (1997). Induction of cell migration by matrix metalloprotease-2 cleavage of laminin-5. *Science*.

[7] Sternlicht MD, Werb Z (2001). How matrix metalloproteinases regulate cell behavior. *Annual Review of Cell and Developmental Biology*.

[8] Egeblad M, Werb Z (2002). New functions for the matrix metalloproteinases in cancer progression. *Nature Reviews Cancer*.

[9] Hadler-Olsen E, Fadnes B, Sylte I, Uhlin-Hansen L, Winberg JO (2011). Regulation of matrix metalloproteinase activity in health and disease. *FEBS Journal*.

[10] Björklund M, Koivunen E (2005). Gelatinase-mediated migration and invasion of cancer cells. *Biochimica et Biophysica Acta*.

[11] Ogata Y, Enghild JJ, Nagase H (1992). Matrix metalloproteinase 3 (stromelysin) activates the precursor for the human matrix metalloproteinase 9. *Journal of Biological Chemistry*.

[12] Zhao YG, Xiao AZ, Newcomer RG (2003). Activation of pro-gelatinase B by endometase/matrilysin-2 promotes invasion of human prostate cancer cells. *Journal of Biological Chemistry*.

[13] Vilen ST, Nyberg P, Hukkanen M (2008). Intracellular co-localization of trypsin-2 and matrix metalloprotease-9: possible proteolytic cascade of trypsin-2, MMP-9 and enterokinase in carcinoma. *Experimental Cell Research*.

[14] Sorsa T, Salo T, Koivunen E (1997). Activation of type IV procollagenases by human tumor-associated trypsin- 2. *Journal of Biological Chemistry*.

[15] Mazzieri R, Masiero L, Zanetta L (1997). Control of type IV collagenase activity by the urokinase-plasmin system: a regulatory mechanism with cell-bound reactants. *EMBO Journal*.

[16] Han YP, Nien YD, Garner WL (2002). Tumor necrosis factor-*α*-induced proteolytic activation of pro-matrix metalloproteinase-9 by human skin is controlled by down-regulating tissue inhibitor of metalloproteinase-1 and mediated by tissue-associated chymotrypsin-like proteinase. *Journal of Biological Chemistry*.

[17] Fridman R, Toth M, Pena D, Mobashery S (1995). Activation of progelatinase B (MMP-9) by gelatinase A (MMP-2). *Cancer Research*.

[18] Von Bredow DC, Cress AE, Howard EW, Bowden GT, Nagle RB (1998). Activation of gelatinase-tissue-inhibitors-of-metalloproteinase complexes by matrilysin. *Biochemical Journal*.

[19] Nakamura H, Fujii Y, Ohuchi E, Yamamoto E, Okada Y (1998). Activation of the precursor of human stromelysin 2 and its interactions with other matrix metalloproteinases. *European Journal of Biochemistry*.

[20] Knäuper V, Smith B, López-Otin C, Murphy G (1997). Activation of progelatinase B (proMMP-9) by active collagenase-3 (MMP-13). *European Journal of Biochemistry*.

[21] Peppin GJ, Weiss SJ (1986). Activation of the endogenous metalloproteinase, gelatinase, by triggered human neutrophils. *Proceedings of the National Academy of Sciences of the United States of America*.

[22] Gu Z, Kaul M, Yan B (2002). S-nitrosylation of matrix metalloproteinases: signaling pathway to neuronal cell death. *Science*.

[23] Bannikov GA, Karelina TV, Collier IE, Marmer BL, Goldberg GI (2002). Substrate binding of gelatinase B induces its enzymatic activity in the presence of intact propeptide. *Journal of Biological Chemistry*.

[24] Graves LE, Ariztia EV, Navari JR, Matzel HJ, Stack MS, Fishman DA (2004). Proinvasive properties of ovarian cancer ascites-derived membrane vesicles. *Cancer Research*.

[25] Schnaeker EM, Ossig R, Ludwig T (2004). Microtubule-dependent matrix metalloproteinase-2/matrix metalloproteinase-9 exocytosis: prerequisite in human melanoma cell invasion. *Cancer Research*.

[26] Hong SD, Hong SP, Lee JI, Lim CY (2000). Expression of matrix metalloproteinase-2 and -9 in oral squamous cell carcinomas with regard to the metastatic potential. *Oral Oncology*.

[27] Brew K, Nagase H (2010). The tissue inhibitors of metalloproteinases (TIMPs): an ancient family with structural and functional diversity. *Biochimica et Biophysica Acta*.

[28] Howard EW, Bullen EC, Banda MJ (1991). Preferential inhibition of 72- and 92-kDa gelatinases by tissue inhibitor of metalloproteinases-2. *Journal of Biological Chemistry*.

[29] O’Connell JP, Willenbrock F, Docherty AJP, Eaton D, Murphy G (1994). Analysis of the role of the COOH-terminal domain in the activation, proteolytic activity, and tissue inhibitor of metalloproteinase interactions of gelatinase B. *Journal of Biological Chemistry*.

[30] Butler GS, Apte SS, Willenbrock F, Murphy G (1999). Human tissue inhibitor of metalloproteinases 3 interacts with both the N- and C-terminal domains of gelatinases A and B: regulation by polyanions. *Journal of Biological Chemistry*.

[31] Liu YE, Wang M, Greene J (1997). Preparation and characterization of recombinant tissue inhibitor of metalloproteinase 4 (TIMP-4). *Journal of Biological Chemistry*.

[32] García MPS, Suárez-Peñaranda JM, Gayoso-Diz P, Barros-Angueira F, Gándara-Rey JM, García-García A (2012). Tissue inhibitor of metalloproteinases in oral squamous cell carcinomas—a therapeutic target?. *Cancer Letters*.

[33] Brown PD (1998). Matrix metalloproteinase inhibitors. *Breast Cancer Research and Treatment*.

[34] Overall CM, Kleifeld O (2006). Towards third generation matrix metalloproteinase inhibitors for cancer therapy. *British Journal of Cancer*.

[35] Wojtowicz-Praga SM, Dickson RB, Hawkins MJ (1997). Matrix metalloproteinase inhibitors. *Investigational New Drugs*.

[36] Zucker S, Cao J, Chen WT (2000). Critical appraisal of the use of matrix metalloproteinase inhibitors in cancer treatment. *Oncogene*.

[37] Coussens LM, Fingleton B, Matrisian LM (2002). Matrix metalloproteinase inhibitors and cancer: trials and tribulations. *Science*.

[38] Koivunen E, Arap W, Valtanen H (1999). Tumor targeting with a selective gelatinase inhibitor. *Nature Biotechnology*.

[39] Heikkilä P, Suojanen J, Pirilä E (2006). Human tongue carcinoma growth is inhibited by selective antigelatinolytic peptides. *International Journal of Cancer*.

[40] Falk V, Soccal PM, Grünenfelder J, Hoyt G, Walther T, Robbins RC (2002). Regulation of matrix metalloproteinases and effect of MMP-inhibition in heart transplant related reperfusion injury. *European Journal of Cardio-Thoracic Surgery*.

[41] Underwood CK, Min D, Lyons JG, Hambley TW (2003). The interaction of metal ions and Marimastat with matrix metalloproteinase 9. *Journal of Inorganic Biochemistry*.

[42] Björklund M, Heikkilä P, Koivunen E (2004). Peptide inhibition of catalytic and noncatalytic activities of matrix metalloproteinase-9 blocks tumor cell migration and invasion. *Journal of Biological Chemistry*.

[43] Sutton TA, Kelly KJ, Mang HE, Plotkin Z, Sandoval RM, Dagher PC (2005). Minocycline reduces renal microvascular leakage in a rat model of ischemic renal injury. *American Journal of Physiology*.

[44] Mirastschijski U, Schnabel R, Claes J, Schneider W, Ågren MS, Tomasek JJ (2010). Matrix metalloproteinase inhibition delays wound healing and blocks the latent transforming growth factor-*β*1-promoted myofibroblast formation and function. *Wound Repair and Regeneration*.

[45] Hirte H, Vergote IB, Jeffrey JR (2006). A phase III randomized trial of BAY 12-9566 (tanomastat) as maintenance therapy in patients with advanced ovarian cancer responsive to primary surgery and paclitaxel/platinum containing chemotherapy: a National Cancer Institute of Canada Clinical Trials Group Study. *Gynecologic Oncology*.

[46] Shalinsky DR, Brekken J, Zou H (1999). Broad antitumor and antiangiogenic activities of AG3340, a potent and selective MMP inhibitor undergoing advanced oncology clinical trials. *Annals of the New York Academy of Sciences*.

[47] Naglich JG, Jure-Kunkel M, Gupta E (2001). Inhibition of angiogenesis and metastasis in two murine models by the matrix metalloproteinase inhibitor, BMS-275291. *Cancer Research*.

[48] Seftor REB, Seftor EA, De Larco JE (1998). Chemically modified tetracyclines inhibit human melanoma cell invasion and metastasis. *Clinical and Experimental Metastasis*.

[49] Sorsa T, Ramamurthy NS, Vernillo AT (1998). Functional sites of chemically modified tetracyclines: inhibition of the oxidative activation of human neutrophil and chicken osteoclast pro-matrix metalloproteinases. *Journal of Rheumatology*.

[50] Golub LM, Sorsa T, Lee HM (1995). Doxycycline inhibits neutrophil (PMN)-type matrix metalloproteinases in human adult periodontitis gingiva. *Journal of Clinical Periodontology*.

[51] Gu Z, Cui J, Brown S (2005). A highly specific inhibitor of matrix metalloproteinase-9 rescues laminin from proteolysis and neurons from apoptosis in transient focal cerebral ischemia. *Journal of Neuroscience*.

[52] Teronen O, Laitinen M, Salo T (2000). Inhibition of matrix metalloproteinases by bisphosphonates may in part explain their effects in the treatment of multiple myeloma. *Blood*.

[53] Gendron R, Grenier D, Sorsa T, Mayrand D (1999). Inhibition of the activities of matrix metalloproteinases 2, 8, and 9 by chlorhexidine. *Clinical and Diagnostic Laboratory Immunology*.

[54] Mitropoulou TN, Tzanakakis GN, Kletsas D, Kalofonos HP, Karamanos NK (2003). Letrozole as a potent inhibitor of cell proliferation and expression of metalloproteinases (MMP-2 and MMP-9) by human epithelial breast cancer cells. *International Journal of Cancer*.

[55] Annabi B, Bouzeghrane M, Currie JC (2005). A PSP94-derived peptide PCK3145 inhibits MMP-9 secretion and triggers CD44 cell surface shedding: implication in tumor metastasis. *Clinical and Experimental Metastasis*.

[56] De Wever O, Mareel M (2003). Role of tissue stroma in cancer cell invasion. *Journal of Pathology*.

[57] Mareel M, Oliveira MJ, Madani I (2009). Cancer invasion and metastasis: interacting ecosystems. *Virchows Archiv*.

[58] Pietras K, Östman A (2010). Hallmarks of cancer: interactions with the tumor stroma. *Experimental Cell Research*.

[59] Impola U, Uitto VJ, Hietanen J (2004). Differential expression of matrilysin-I (MMP-7), 92 kD gelatinase (MMP-9), and metalloelastase (MMP-12) in oral verrucous and squamous cell cancer. *Journal of Pathology*.

[60] Sutinen M, Kainulainen T, Hurskainen T (1998). Expression of matrix metalloproteinases (MMP-1 and -2) and their inhibitors (TIMP-1, -2 and -3) in oral lichen planus, dysplasia, squamous cell carcinoma and lymph node metastasis. *British Journal of Cancer*.

[61] Fullár A, Kovalszky I, Bitsche M (2012). Tumor cell and carcinoma-associated fibroblast interaction regulates matrix metalloproteinases and their inhibitors in oral squamous cell carcinoma. *Experimental Cell Research*.

[62] Nurmenniemi S, Sinikumpu T, Alahuhta I (2009). A novel organotypic model mimics the tumor microenvironment. *American Journal of Pathology*.

[63] Suojanen J, Vilen S-T, Nyberg P (2011). Selective gelatinase inhibitor peptide is effective in targeting tongue carcinoma cell tumors In Vivo. *Anticancer Research*.

[64] Wolf K, Mazo I, Leung H (2003). Compensation mechanism in tumor cell migration: mesenchymal-amoeboid transition after blocking of pericellular proteolysis. *Journal of Cell Biology*.

[65] Gearing AJH, Beckett P, Christodoulou M (1995). Matrix metalloproteinases and processing of pro-TNF-*α*. *Journal of Leukocyte Biology*.

[66] Ito A, Mukaiyama A, Itoh Y (1996). Degradation of interleukin 1*β* by matrix metalloproteinases. *Journal of Biological Chemistry*.

[67] Van Den Steen PE, Proost P, Wuyts A, Van Damme J, Opdenakker G (2000). Neutrophil gelatinase B potentiates interleukin-8 tenfold by aminoterminal processing, whereas it degrades CTAP-III, PF-4, and GRO-*α* and leaves RANTES and MCP-2 intact. *Blood*.

[68] Ferreras M, Felbor U, Lenhard T, Olsen BR, Delaissé JM (2000). Generation and degradation of human endostatin proteins by various proteinases. *FEBS Letters*.

[69] Heljasvaara R, Nyberg P, Luostarinen J (2005). Generation of biologically active endostatin fragments from human collagen XVIII by distinct matrix metalloproteases. *Experimental Cell Research*.

[70] Kurahara S-I, Shinohara M, Ikebe T (1999). Expression of MMPs, MT-MMP, and TIMPs in squamous cell carcinoma of the oral cavity: correlations with tumor invasion and metastasis. *Head and Neck*.

[71] Zhou CX, Gao Y, Johnson NW, Gao J (2010). Immunoexpression of matrix metalloproteinase-2 and matrix metalloproteinase-9 in the metastasis of squamous cell carcinoma of the human tongue. *Australian Dental Journal*.

[72] Yorioka CW, Coletta RD, Alves F, Nishimoto IN, Kowalski LP, Graner E (2002). Matrix metalloproteinase-2 and -9 activities correlate with the disease-free survival of oral squamous cell carcinoma patients. *International Journal of Oncology*.

[73] Guttman D, Stern Y, Shpitzer T, Ulanovski D, Druzd T, Feinmesser R (2004). Expression of MMP-9, TIMP-1, CD-34 and factor-8 as prognostic markers for squamous cell carcinoma of the tongue. *Oral Oncology*.

[74] Katayama A, Bandoh N, Kishibe K (2004). Expressions of matrix metalloproteinases in early-stage oral squamous cell carcinoma as predictive indicators for tumor metastases and prognosis. *Clinical Cancer Research*.

[75] Ikebe T, Shinohara M, Takcuchi H (1999). Gelatinolytic activity of matrix metalloproteinase in tumor tissues correlates with the invasiveness of oral cancer. *Clinical and Experimental Metastasis*.

[76] Kato K, Hara A, Kuno T (2005). Matrix metalloproteinases 2 and 9 in oral squamous cell carcinomas: manifestation and localization of their activity. *Journal of Cancer Research and Clinical Oncology*.

[77] Lin C-W, Tseng S-W, Yang S-F (2012). Role of lipocalin 2 and its complex with matrix metalloproteinase-9 in oral cancer. *Oral Diseases*.

[78] De Vicente JC, Fresno MF, Villalain L, Vega JA, Hernández Vallejo G (2005). Expression and clinical significance of matrix metalloproteinase-2 and matrix metalloproteinase-9 in oral squamous cell carcinoma. *Oral Oncology*.

[79] Stokes A, Joutsa J, Ala-aho R (2010). Expression profiles and clinical correlations of degradome components in the tumor microenvironment of head and neck squamous cell carcinoma. *Clinical Cancer Research*.

[80] Fan H-X, Li H-X, Gao Z-X, Zheng J-H (2012). Changes in the expression of MMP2, MMP9, and ColIV in stromal cells in oral squamous tongue cell carcinoma: relationships and prognostic implications. *Journal of Experimental and Clinical Cancer Research*.

[81] Siewko M, Mroczko B, Szmitkowski M (2012). The role of matrix metalloproteinases (MMPs) and their inhibitors (TIMPs) in the development of esophageal cancer. *Folia Histochemica et Cytobiologica*.

[82] Hu X, Li D, Zhang W, Zhou J, Tang B (2012). Matrix metalloproteinase-9 expression correlates with prognosis and involved in ovarian cancer cell invasion. *Archives of Gynecology and Obstetrics*.

[83] Zhang QW, Liu L, Chen R (2012). Matrix metalloproteinase-9 as a prognostic factor in gastric cancer: a meta-analysis. *Asian Pacific Journal of Cancer Prevention*.

[84] Ruokolainen H, Pääkkö P, Turpeenniemi-Hujanen T (2005). Serum matrix metalloproteinase-9 in head and neck squamous cell carcinoma is a prognostic marker. *International Journal of Cancer*.

[85] Stetler-Stevenson WG, Aznavoorian S, Liotta LA (1993). Tumor cell interactions with the extracellular matrix during invasion and metastasis. *Annual Review of Cell Biology*.

[86] Patterson BC, Sang QA (1997). Angiostatin-converting enzyme activities of human matrilysin (MMP-7) and gelatinase B/type IV collagenase (MMP.9). *Journal of Biological Chemistry*.

[87] Bergers G, Brekken R, McMahon G (2000). Matrix metalloproteinase-9 triggers the angiogenic switch during carcinogenesis. *Nature Cell Biology*.

[88] Mantovani A (2005). Cancer: inflammation by remote control. *Nature*.

[89] Aggarwal BB, Shishodia S, Sandur SK, Pandey MK, Sethi G (2006). Inflammation and cancer: how hot is the link?. *Biochemical Pharmacology*.

[90] Ribatti D, Crivellato E (2012). Mast cells, angiogenesis, and tumour growth. *Biochimica et Biophysica Acta*.

[91] Sun L, Diamond ME, Ottaviano AJ, Joseph MJ, Ananthanarayan V, Munshi HG (2008). Transforming growth factor-*β*1 promotes matrix metalloproteinase-9- mediated oral cancer invasion through snail expression. *Molecular Cancer Research*.

[92] Takayama S, Hatori M, Kurihara Y, Kinugasa Y, Shirota T, Shintai S (2009). Inhibition of TGF-*β*1 suppresses motility and invasiveness of oral squamous cell carcinoma cell lines via modulation of integrins and down-regulation of matrix-metalloproteinases. *Oncology Reports*.

[93] Yu Q, Stamenkovic I (2000). Cell surface-localized matrix metalloproteinase-9 proteolytically activates TGF-*β* and promotes tumor invasion and angiogenesis. *Genes and Development*.

[94] Shchors K, Evan G (2007). Tumor angiogenesis: cause or consequence of cancer?. *Cancer Research*.

[95] Sommer G, Weise S, Kralisch S (2009). Lipocalin-2 is induced by interleukin-1 *β* in murine adipocytes in vitro. *Journal of Cellular Biochemistry*.

[96] Watanabe H, Iwase M, Ohashi M, Nagumo M (2002). Role of interleukin-8 secreted from human oral squamous cell carcinoma cell lines. *Oral Oncology*.

[97] Chakrabarti S, Patel KD (2005). Regulation of matrix metalloproteinase-9 release from IL-8-stimulated human neutrophils. *Journal of Leukocyte Biology*.

[98] Rao SK, Pavicevic Z, Du Z (2010). Pro-inflammatory genes as biomarkers and therapeutic targets in oral squamous cell carcinoma. *Journal of Biological Chemistry*.

[99] Ishikawa T, Nakashiro KI, Hara S (2006). CXCR4 expression is associated with lymph-node metastasis of oral squamous cell carcinoma. *International Journal of Oncology*.

[100] Lee JI, Jin BH, Kim MA, Yoon HJ, Hong SP, Hong SD (2009). Prognostic significance of CXCR-4 expression in oral squamous cell carcinoma. *Oral Surgery, Oral Medicine, Oral Pathology, Oral Radiology and Endodontology*.

[101] Chan G, Boyle JO, Yang EK (1999). Cyclooxygenase-2 expression is up-regulated in squamous cell carcinoma of the head and neck. *Cancer Research*.

[102] Kwak YE, Jeon NK, Kim J, Eun JL (2007). The cyclooxygenase-2 selective inhibitor celecoxib suppresses proliferation and invasiveness in the human oral squamous carcinoma. *Annals of the New York Academy of Sciences*.

[103] Hanahan D, Weinberg RA (2011). Hallmarks of cancer: the next generation. *Cell*.

[104] Hanahan D, Weinberg RA (2000). The hallmarks of cancer. *Cell*.

[105] Homer JJ, Greenman J, Stafford ND (2000). Angiogenesis in head and neck squamous cell carcinoma. *Clinical Otolaryngology and Allied Sciences*.

[106] Roodink I, Leenders WPJ (2010). Targeted therapies of cancer: angiogenesis inhibition seems not enough. *Cancer Letters*.

[107] Mǎrgǎritescu C, Pirici D, Simionescu C (2009). VEGF and VEGFRs expression in oral squamous cell carcinoma. *Romanian Journal of Morphology and Embryology*.

[108] Shweiki D, Neeman M, Itin A, Keshet E (1995). Induction of vascular endothelial growth factor expression by hypoxia and by glucose deficiency in multicell spheroids: implications for tumor angiogenesis. *Proceedings of the National Academy of Sciences of the United States of America*.

[109] Stewart J, Siavash H, Hebert C, Norris K, Nikitakis NG, Sauk JJ (2003). Phenotypic switching of VEGF and collagen XVIII during hypoxia in head and neck squamous carcinoma cells. *Oral Oncology*.

[110] Olsson AK, Dimberg A, Kreuger J, Claesson-Welsh L (2006). VEGF receptor signalling—in control of vascular function. *Nature Reviews Molecular Cell Biology*.

[111] Forsythe JA, Jiang BH, Iyer NV (1996). Activation of vascular endothelial growth factor gene transcription by hypoxia-inducible factor 1. *Molecular and Cellular Biology*.

[112] Liu CJ, Chang KW, Lin SC, Cheng HW (2009). Presurgical serum levels of matrix metalloproteinase-9 and vascular endothelial growth factor in oral squamous cell carcinoma. *Oral Oncology*.

[113] Osinsky SP, Ganusevich II, Bubnovskaya LN (2005). Hypoxia level and matrix metalloproteinases-2 and -9 activity in lewis lung carcinoma: correlation with metastasis. *Experimental Oncology*.

[114] Hiratsuka S, Nakamura K, Iwai S (2002). MMP9 induction by vascular endothelial growth factor receptor-1 is involved in lung-specific metastasis. *Cancer Cell*.

[115] Henriques AC, de Matos FR, Galvão HC, Freitas RA (2012). Immunohistochemical expression of MMP-9 and VEGF in squamous cell carcinoma of the tongue. *Journal of Oral Science*.

[116] Kim SH, Kim K, Lee JS, Koo BS, Kim JH, Choi EC (2006). Correlations of oral tongue cancer invasion with matrix metalloproteinases (MMPs) and vascular endothelial growth factor (VEGF) expression. *Journal of Surgical Oncology*.

[117] Nikitakis NG, Rivera H, Lopes MA (2003). Immunohistochemical expression of angiogenesis-related markers in oral squamous cell carcinomas with multiple metastatic lymph nodes. *American Journal of Clinical Pathology*.

[118] Väänänen A, Ylipalosaari M, Parikka M (2007). Collagen XVIII modulation is altered during progression of oral dysplasia and carcinoma. *Journal of Oral Pathology and Medicine*.

[119] Homer JJ, Greenman J, Stafford ND (2002). Circulating angiogenic cytokines as tumour markers and prognostic factors in head and neck squamous cell carcinoma. *Clinical Otolaryngology and Allied Sciences*.

[120] Nyberg P, Heikkilä P, Sorsa T (2003). Endostatin inhibits human tongue carcinoma cell invasion and intravasation and blocks the activation of matrix metalloprotease-2, -9, and -13. *Journal of Biological Chemistry*.

[121] Peng F, Xu Z, Wang J (2012). Recombinant human endostatin normalizes tumor vasculature and enhances radiation response in xenografted human nasopharyngeal carcinoma models. *PLoS ONE*.

[122] O’Reilly MS, Holmgren L, Shing Y (1994). Angiostatin: a novel angiogenesis inhibitor that mediates the suppression of metastases by a Lewis lung carcinoma. *Cell*.

[123] Pozzi A, LeVine WF, Gardner HA (2002). Low plasma levels of matrix metalloproteinase 9 permit increased tumor angiogenesis. *Oncogene*.

[124] Matsumoto G, Ohmi Y, Shindo J (2001). Angiostatin gene therapy inhibits the growth of murine squamous cell carcinoma in vivo. *Oral Oncology*.

[125] Hamano Y, Zeisberg M, Sugimoto H (2003). Physiological levels of tumstatin, a fragment of collagen IV *α*3 chain, are generated by MMP-9 proteolysis and suppress angiogenesis via *α*V*β*3 integrin. *Cancer Cell*.

[126] Maeshima Y, Manfredi M, Reimerli C (2001). Identification of the anti-angiogenic site within vascular basement membrane-derived tumstatin. *Journal of Biological Chemistry*.

[127] Choi S, Myers JN (2008). Molecular pathogenesis of oral squamous cell carcinoma: implications for therapy. *Journal of Dental Research*.

[128] Walker DM, Boey G, McDonald LA (2003). The pathology of oral cancer. *Pathology*.

[129] Van Oijen MGCT, Slootweg PJ (2000). Oral field cancerization: carcinogen-induced independent events or micrometastatic deposits?. *Cancer Epidemiology Biomarkers and Prevention*.

[130] Vairaktaris E, Vassiliou S, Nkenke E (2008). A metalloproteinase-9 polymorphism which affects its expression is associated with increased risk for oral squamous cell carcinoma. *European Journal of Surgical Oncology*.

[131] Tamamura R, Nagatsuka H, Siar CH Comparative analysis of basal lamina type IV collagen alpha chains, matrix metalloproteinases-2 and -9 expressions in oral dysplasia and invasive carcinoma.

[132] Jordan RCK, Macabeo-Ong M, Shiboski CH (2004). Overexpression of matrix metalloproteinase-1 and -9 mRNA is associated with progression of oral dysplasia to cancer. *Clinical Cancer Research*.

[133] Ogbureke KU, Weinberger PM, Looney SW (2012). Expressions of matrix metalloproteinase-9 (MMP-9), dentin sialophosphoprotein (DSPP), and osteopontin (OPN) at histologically negative surgical margins may predict recurrence of oral squamous cell carcinoma. *Oncotarget*.

[134] Coussens LM, Tinkle CL, Hanahan D, Werb Z (2000). MMP-9 supplied by bone marrow-derived cells contributes to skin carcinogenesis. *Cell*.

[135] Ramqvist T, Dalianis T (2010). Oropharyngeal cancer epidemic and human papillomavirus. *Emerging Infectious Diseases*.

[136] Andreoli MA, di Loreto C, Filho AL, Villa LL, Maria-Engler SS (2012). HPV16 oncoproteins induce MMPs/RECK-TIMP-2 imbalance in primary keratinocytes: possible implications in cervical carcinogenesis. *PLoS ONE*.

[137] Vilen S-T, Suojanen J, Salas F (2012). Trypsin-2 enhances carcinoma invasion by processing tight junctions and activating promt1-mmp. *Cancer Investigation*.

[138] Pirilä E, Maisi P, Salo T, Koivunen E, Sorsa T (2001). In vivo localization of gelatinases (MMP-2 and -9) by in situ zymography with a selective gelatinase inhibitor. *Biochemical and Biophysical Research Communications*.

[139] Snoek-van Beurden PAM, Von Den Hoff JW (2005). Zymographic techniques for the analysis of matrix metalloproteinases and their inhibitors. *BioTechniques*.

[140] Jin L, Lloyd RV (1997). In situ hybridization: methods and applications. *Journal of Clinical Laboratory Analysis*.

[141] Singh RD, Haridas N, Patel JB (2010). Matrix metalloproteinases and their inhibitors: correlation with invasion and metastasis in oral cancer. *Indian Journal of Clinical Biochemistry*.

[142] Patel BP, Shah SV, Shukla SN, Shah PM, Patel PS (2007). Clinical significance of MMP-2 and MMP-9 in patients with oral cancer. *Head and Neck*.

[143] Berg JM, Tymoczko JL, Stryer L (2002). *Biochemistry*.

[144] Suojanen J, Salo T, Koivunen E, Sorsa T, Pirilä E (2009). A novel and selective membrane type-1 matrix metalloproteinase (MT1-MMp) inhibitor reduces cancer cell motility and tumor growth. *Cancer Biology and Therapy*.

[145] Hotary K, Li XY, Allen E, Stevens SL, Weiss SJ (2006). A cancer cell metalloprotease triad regulates the basement membrane transmigration program. *Genes and Development*.

[146] Luukkaa H, Klemi P, Leivo I (2010). Expression of matrix metalloproteinase-1, -7, -9, -13, Ki-67, and HER-2 in epithelial-myoepithelial salivary gland cancer. *Head and Neck*.

[147] Bendrik C, Robertson J, Gauldie J, Dabrosin C (2008). Gene transfer of matrix metalloproteinase-9 induces tumor regression of breast cancer in vivo. *Cancer Research*.

[148] Garg P, Sarma D, Jeppsson S (2010). Matrix metalloproteinase-9 functions as a tumor suppressor in colitis-associated cancer. *Cancer Research*.

